# Supplementary material for: Characterization of Microbial Diversity of Two Tomato Cultivars through Targeted Next-Generation Sequencing 16S rRNA and ITS Techniques
Source: Microorganisms. 2023 Sep 18;11(9):2337. doi: 10.3390/microorganisms11092337 (PMC10534366; doi:10.3390/microorganisms11092337)
Supplement: Supplementary file 1 [file microorganisms-11-02337-s001.zip › Figures.pptx]

## Slide 1
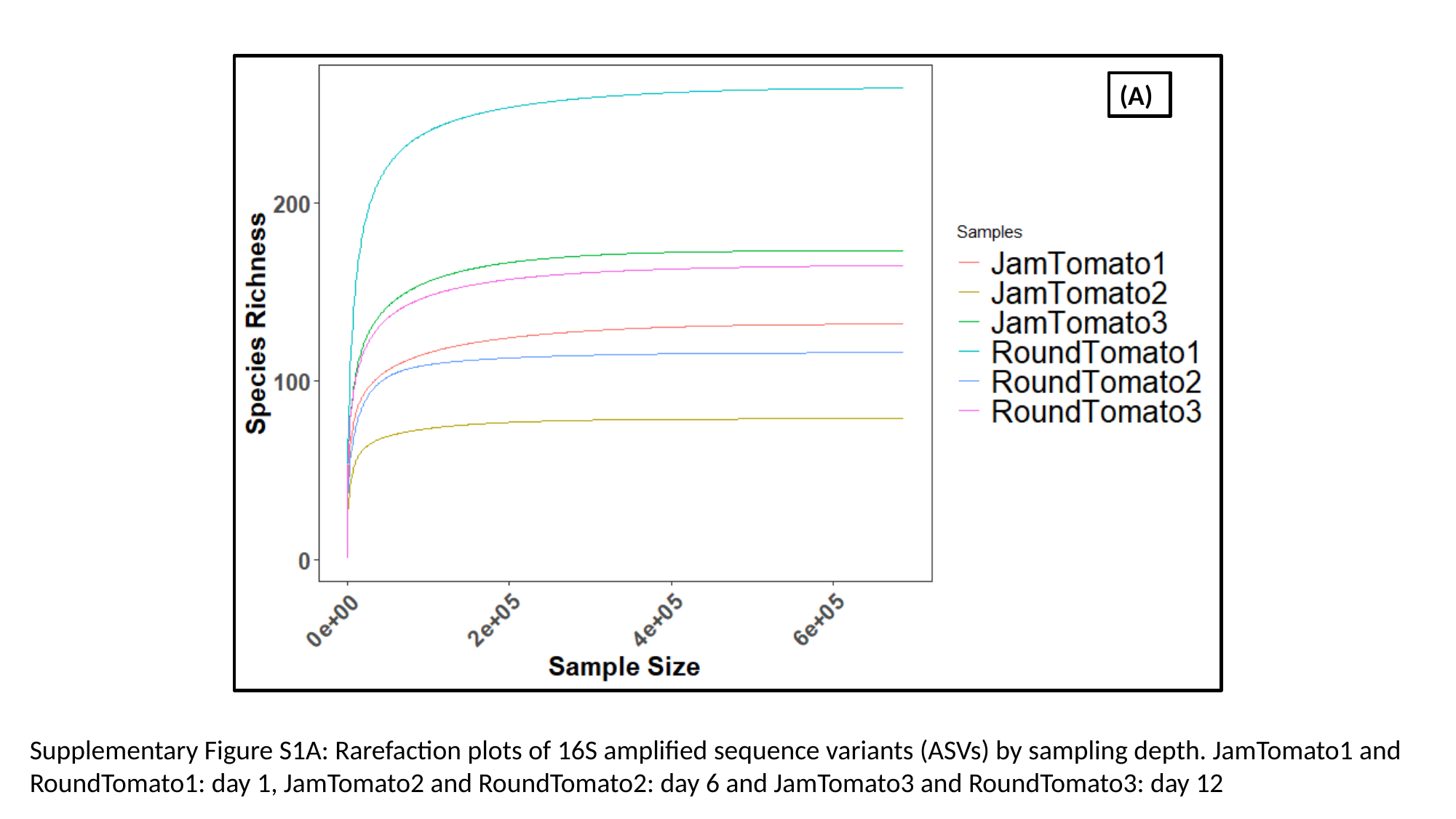

(A)
Supplementary Figure S1A: Rarefaction plots of 16S amplified sequence variants (ASVs) by sampling depth. JamTomato1 and RoundTomato1: day 1, JamTomato2 and RoundTomato2: day 6 and JamTomato3 and RoundTomato3: day 12

## Slide 2
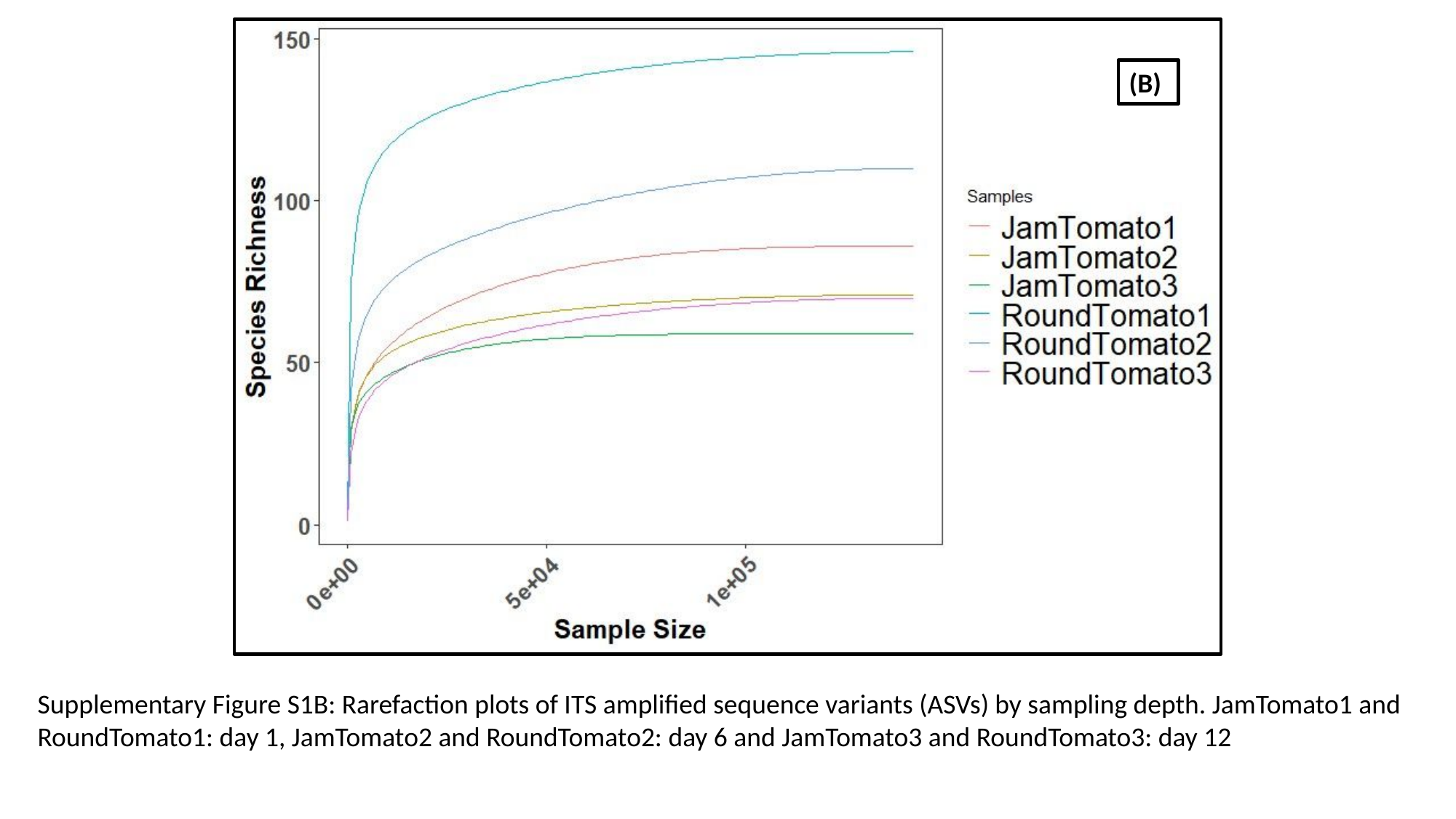

(B)
Supplementary Figure S1B: Rarefaction plots of ITS amplified sequence variants (ASVs) by sampling depth. JamTomato1 and RoundTomato1: day 1, JamTomato2 and RoundTomato2: day 6 and JamTomato3 and RoundTomato3: day 12

## Slide 3
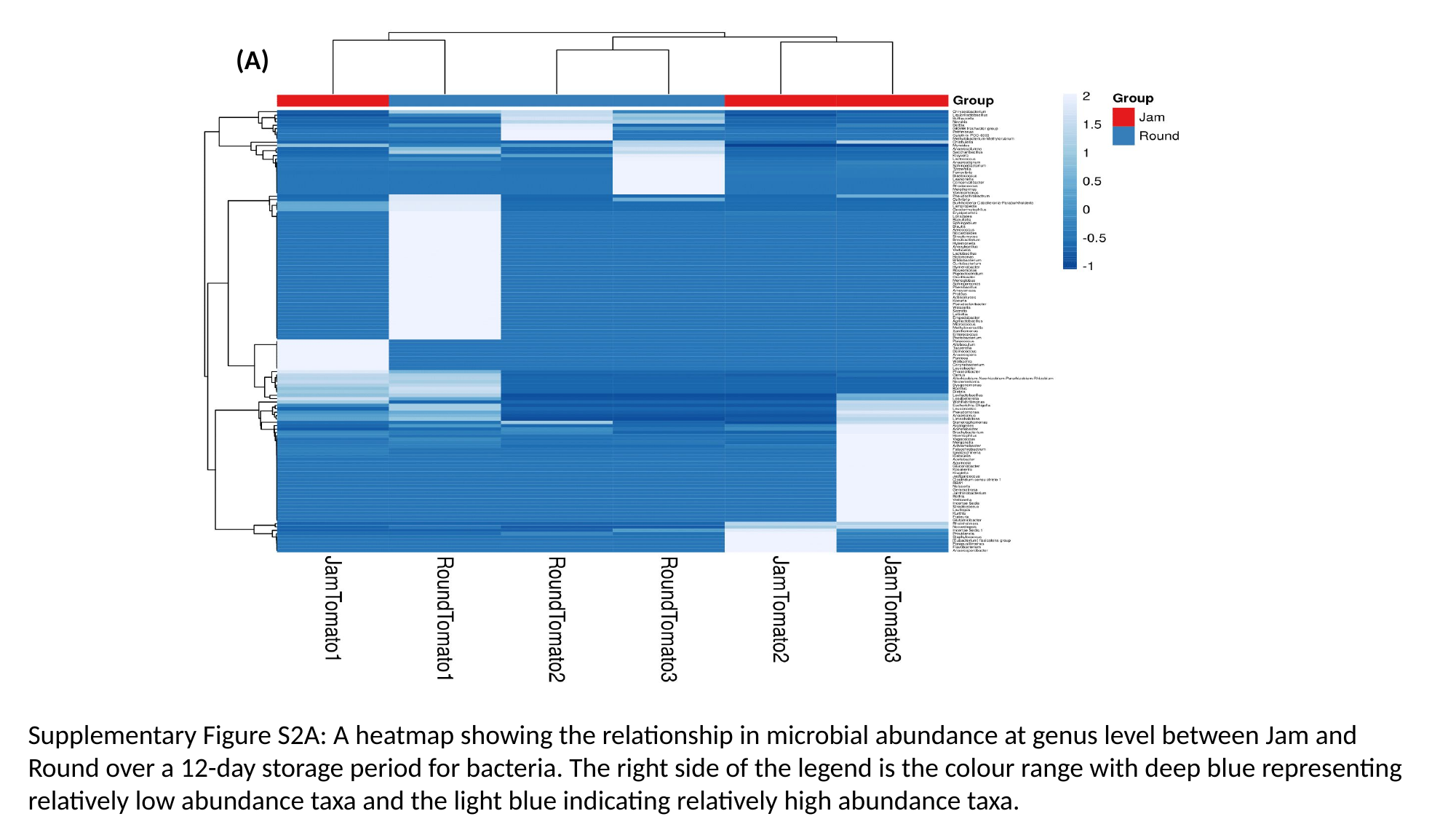

(A)
Supplementary Figure S2A: A heatmap showing the relationship in microbial abundance at genus level between Jam and Round over a 12-day storage period for bacteria. The right side of the legend is the colour range with deep blue representing relatively low abundance taxa and the light blue indicating relatively high abundance taxa.

## Slide 4
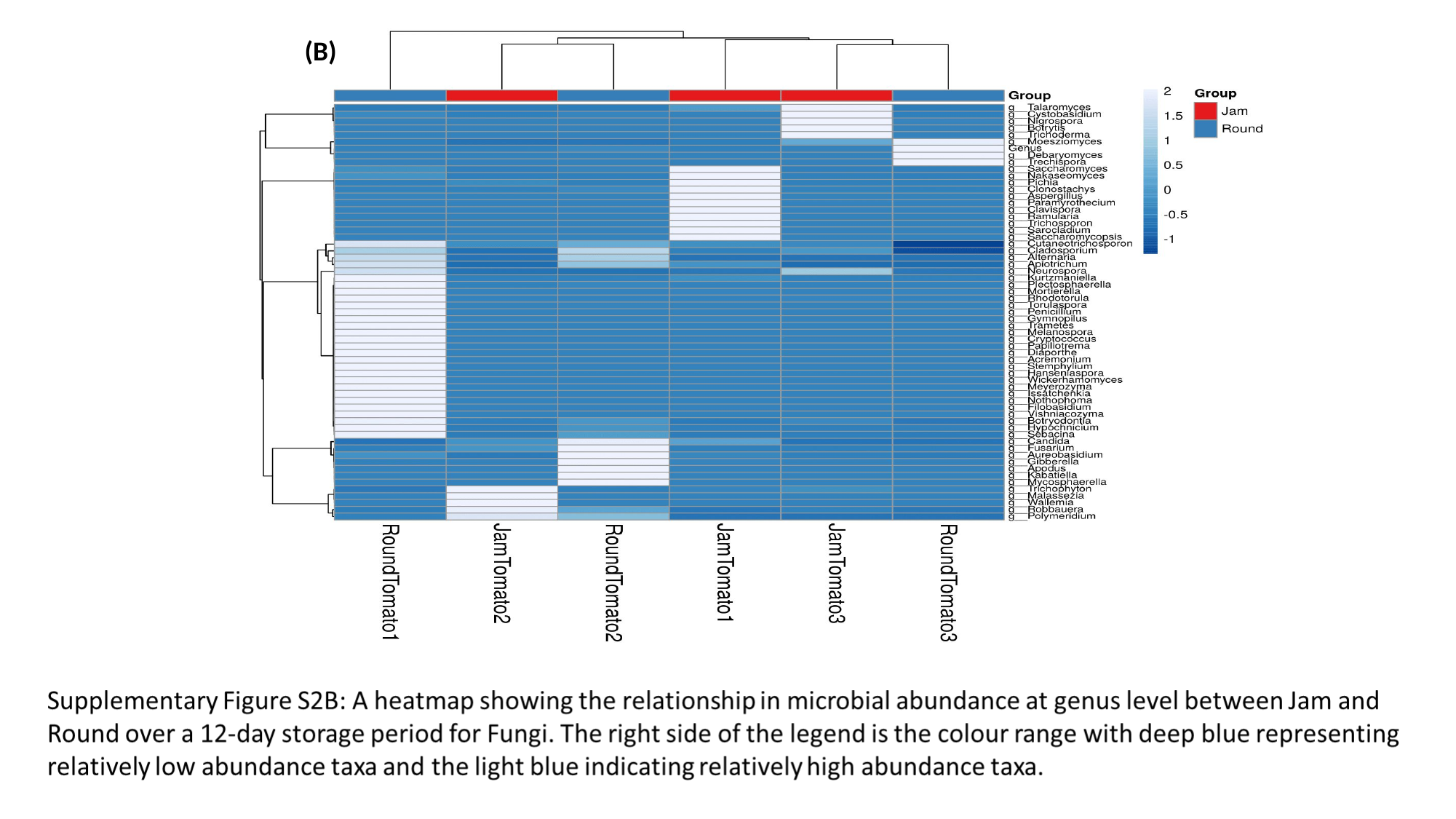

(B)
